# Prognostic Nutritional Index as a Predictor of Complications Following Robot-Assisted Radical Cystectomy

**DOI:** 10.7759/cureus.84470

**Published:** 2025-05-20

**Authors:** Masayuki Kurokawa, Toru Sugihara, Risako Watanabe, Hayato Hoshina, Eiichiro Takaoka, Satoshi Ando, Jun Kamei, Tetsuya Fujimura

**Affiliations:** 1 Department of Urology, Jichi Medical University, Shimotsuke, JPN; 2 Department of Urology, The University of Tokyo Graduate School of Medicine, Tokyo, JPN

**Keywords:** bladder cancer, postoperative complications, postoperative ileus, prognostic nutritional index, robot-assisted radical cystectomy

## Abstract

Objective

The objective of this study is to assess how the preoperative prognostic nutritional index (PNI) affects the occurrence of postoperative complications in patients undergoing robot-assisted radical cystectomy (RARC).

Methods

We retrospectively analyzed data from 103 patients who underwent RARC at Jichi Medical University Hospital between June 2018 and December 2023. The PNI was calculated using the following formula: 10 × serum albumin + 0.005 × total lymphocyte count. Patients were divided into high- and low-PNI groups based on a threshold value of 45. Postoperative complications occurring within 30 days were compared between the two groups, and risk factors were identified using multivariate logistic regression analysis.

Results

Postoperative complications occurred in 50 patients (48.5%), with eight patients (8%) experiencing severe complications (Clavien-Dindo Grade ≥3). The most common complication was postoperative ileus, affecting 26% of patients (n = 28). The low-PNI group had significantly higher rates of overall complications (70% vs. 47%, p< 0.05) and postoperative ileus (48% vs. 18%, p < 0.05) compared to the high-PNI group. Multivariate analysis identified low PNI (ORs: 3.82 for overall complications and 3.90 for ileus) and intestinal urinary diversion (ORs: 3.33 and 5.34, respectively) as independent risk factors.

Conclusions

The preoperative PNI is a significant predictor of both overall complications and postoperative ileus following RARC. These findings underscore the importance of preoperative immunonutritional assessment for risk stratification and suggest that nutritional screening and immunonutritional interventions may enhance postoperative outcomes in high-risk patients.

## Introduction

Robot-assisted radical cystectomy (RARC) is increasingly being adopted as a standard therapeutic option [1]. Compared to open radical cystectomy, RARC offers several benefits, including fewer severe complications and surgical site infections (SSIs), reduced blood loss, and comparable oncological outcomes [2,3]. Despite these advantages, the 30-day postoperative complication rate associated with RARC remains high, with an overall rate of 45.7%, including 28% classified as high-grade complications (Clavien-Dindo (CD) Grade III or higher) [1]. This highlights the substantial risk of postoperative complications following RARC for bladder cancer, affecting nearly half of all patients.

Nutritional and immunological status are recognized as critical factors in postoperative recovery. Malnutrition and immunosuppression can delay wound healing, impair host defense mechanisms, and increase the risk of infections and other complications. Therefore, evaluating these factors preoperatively may help identify high-risk patients and enable more individualized perioperative care [4].

The prognostic nutritional index (PNI) has emerged as a useful immunonutritional marker in surgical oncology and has shown promise as a predictor of postoperative complications across various malignancies [4-6]. Calculated from serum albumin levels and total lymphocyte counts obtained during preoperative assessment, the PNI offers an integrated measure of a patient’s nutritional and immune status. While its predictive value has been demonstrated in several types of cancer surgeries, its specific role in forecasting complications following RARC remains underexplored.

This study aimed to examine the relationship between preoperative PNI and the incidence of postoperative complications in patients undergoing RARC for bladder cancer. Additionally, we sought to determine whether incorporating PNI into routine preoperative evaluation could enhance risk stratification and support the implementation of targeted perioperative interventions.

## Materials and methods

Study design and patient selection

This retrospective study analyzed data from 103 patients who underwent RARC for bladder cancer at Jichi Medical University Hospital between June 2018 and December 2023. The study was approved by the Institutional Ethics Committee of Jichi Medical University Hospital (approval number A22-023). Due to the retrospective nature of the study, the requirement for individual informed consent was waived, and an opt-out approach was implemented to ensure transparency and respect for patient autonomy.

Data collection and variable definitions

The PNI was calculated using preoperative blood test results based on the following formula: 10 × serum albumin (g/dL) + 0.005 × total lymphocyte count (/mm³) [4]. Patients were categorized into high- and low-PNI groups using a validated cutoff value of 45 [7,8], which has been associated with an increased risk of postoperative complications and adverse outcomes in prior surgical oncology research. This threshold is widely recognized as clinically meaningful for risk stratification.

Additional variables collected included age, sex, BMI, Charlson Comorbidity Index (CCI) [9], American Society of Anesthesiologists Physical Status (ASA-PS) classification [10], clinical T and N stages, history of abdominal surgery, neoadjuvant chemotherapy (NAC), intestinal urinary diversion, and simultaneous nephroureterectomy. Age was dichotomized at a cutoff of 70 years.

Surgical procedure

All surgeries were performed using the da Vinci Surgical System (Intuitive Surgical, Inc., Sunnyvale, CA, USA). Patients were positioned at a 25° Trendelenburg angle during RARC, which was adjusted to 15° for intracorporeal urinary diversion (ICUD). Pelvic lymph node dissection routinely included external, internal, and obturator lymph nodes.

Following cystectomy, urinary diversion was achieved via ileal conduit or orthotopic neobladder, depending on renal function, oncological considerations, and patient preference. In selected cases, cutaneous ureterostomy was performed instead of ICUD. Ureterointestinal anastomoses were created using the Bricker technique with running 3-0 Monocryl sutures. Double-J stents (6 Fr) were inserted through the assistant port and typically removed within seven days postoperatively. In cases involving complete urinary tract resection, urinary diversion was omitted. The surgical technique adhered to established institutional protocols and has been previously described by our group [2,3].

Outcome measures

Postoperative complications occurring within 30 days of surgery were recorded and graded using the CD classification system [11]. Complications classified as Grades 1 and 2 were considered low-grade, while Grade ≥3 was defined as high-grade. Primary outcomes included the incidence of all complications, high-grade complications, postoperative ileus, and infections. Postoperative ileus was defined according to standardized criteria [12], while infections included symptomatic UTIs, SSIs, and catheter-related bloodstream infections, as defined by the CDC guidelines [13-15].

Statistical analysis

Continuous variables were expressed as medians with IQRs, while categorical variables were compared using Fisher’s exact test. Univariate analyses were conducted to identify potential predictors of postoperative complications. Variables with p < 0.10 in univariate analysis were included in a multivariate logistic regression model. A p-value of < 0.05 was considered statistically significant. All statistical analyses were performed using EZR software [16], a graphical user interface for R.

## Results

Patient characteristics

The characteristics of the 103 patients are summarized in Table 1. Compared to the high-PNI group, the low-PNI group had a higher proportion of female patients and generally exhibited a lower BMI. In addition, serum albumin levels and lymphocyte counts were lower in the low-PNI group, reflecting a compromised immunonutritional status.

**Table 1:** Patient characteristics Continuous variables are presented as median (IQR). ASA-PS, American Society of Anesthesiologists Physical Status; CCI, Charlson Comorbidity Index; NAC, neoadjuvant chemotherapy; PNI, prognostic nutritional index

Variable	Total (n = 103), n (%) or median (IQR)	PNI ≥45 (n = 76), n (%) or median (IQR)	PNI <45 (n = 27), n (%) or median (IQR)
Age, years	71 (66-75)	69.5 (64.0-74.3)	74.0 (71.5-76.5)
Sex			
Male	80 (78%)	63 (83%)	17 (63%)
Female	23 (22%)	13 (17%)	10 (37%)
BMI, kg/m²	23.1 (21.1-24.9)	23.8 (22.1-25.4)	21.6 (19.2-23.3)
CCI	1 (0-2)	1 (0-1)	1 (0-2)
ASA-PS			
1	4 (4%)	4 (5%)	0 (0%)
2	80 (78%)	58 (76%)	22 (81%)
3	19 (18%)	14 (18%)	5 (19%)
Previous abdominal surgery	29 (28%)	25 (33%)	4 (15%)
NAC	80 (78%)	62 (82%)	18 (67%)
Clinical T stage			
Ta, T1, or Tis	38 (37%)	28 (37%)	10 (37%)
T2	39 (38%)	33 (43%)	6 (22%)
T3 or higher	26 (25%)	15 (20%)	11 (41%)
Clinical N positive	6 (6%)	5 (7%)	1 (4%)
Preoperative laboratory test			
WBC, /mm³	6,100 (5,100-7,350)	6,250 (5,300-7,500)	6,000 (4,200-6,650)
Lymphocyte, /mm³	1,400 (1,150-1,800)	1,600 (1,300-1,900)	1,100 (900-1,250)
Hemoglobin, g/dL	11.4 (10.4-12.7)	11.7 (10.4-13.0)	10.8 (10.3-11.5)
Albumin, g/dL	4.0 (3.8-4.2)	4.1 (3.9-4.2)	3.7 (3.5-3.8)
PNI	47 (44.5-49.5)	48 (46.5-50.5)	42.5 (40.8-43.5)

In terms of tumor progression, stage T3 or higher disease was observed in 15 patients (20%) in the high-PNI group and in 11 patients (41%) in the low-PNI group, indicating a trend toward more advanced disease in the low-PNI group.

Surgical outcomes

The surgical outcomes are summarized in Table 2. Overall, the low-PNI group had a shorter operative time compared to the high-PNI group. Although estimated intraoperative blood loss did not differ significantly between the groups, the low-PNI group required significantly more intraoperative blood transfusions. Despite these differences, the length of hospital stay was comparable between the two groups, with no significant variation observed.

**Table 2:** Surgical outcomes A p-value < 0.05 was considered statistically significant. LOS, length of stay; PNI, prognostic nutritional index

Variable	Total (n = 103), n (%) or median (IQR)	PNI ≥45 (n = 76), n (%) or median (IQR)	PNI <45 (n = 27), n (%) or median (IQR)	p-value
Operative time (minutes)	475 (409-534)	485.5 (425.8-543.8)	462 (402.5-486)	0.051
Estimated blood loss (mL)	300 (145-450)	300 (135-466)	300 (155-550)	0.93
Blood transfusion (intraoperative)	7 (7%)	2 (3%)	5 (19%)	0.01
Urinary diversion type				
Ileal conduit	79 (77%)	60 (79%)	19 (70%)	-
Cutaneous ureterostomy	15 (15%)	12 (16%)	3 (11%)	-
Neobladder	3 (3%)	1 (1%)	2 (7%)	-
None	6 (6%)	3 (4%)	3 (11%)	-
Intestinal urinary diversion	82 (80%)	63 (83%)	21 (78%)	0.79
Simultaneous nephroureterectomy	14 (14%)	10 (13%)	4 (15%)	1
LOS (days)	23 (19.5-28)	22 (19-27)	25 (20-30)	0.28

Complications

Postoperative complications within 30 days are summarized in Table 3. Overall, 48.5% of patients (n = 50) experienced postoperative complications, with 8% (n = 8) classified as severe (Grade ≥3) according to the CD classification. The most common complication was postoperative ileus, occurring in 26% of patients (n = 27), followed by UTIs, reported in 12% (n = 12).

**Table 3:** Perioperative complications according to CD classification CD, Clavien-Dindo; CRBSI, catheter-related bloodstream infection; SSI, surgical site infection

Type of complications	Complication	All complications (CD I-V), n (%)	High-grade (CD III-V), n (%)
Gastrointestinal	Postoperative ileus	27 (26%)	3 (3%)
	Acute cholangitis	1 (1%)	-
	Acute cholecystitis	1 (1%)	-
	Clostridium difficile colitis	1 (1%)	-
Infectious	UTI	12 (12%)	1 (1%)
	Pelvic abscess	3 (3%)	1 (1%)
	CRBSI	2 (2%)	-
	Epididymitis	2 (2%)	-
	SSI	2 (2%)	1 (1%)
	Peritonitis	1 (1%)	-
Cardiovascular	Deep vein thrombosis	2 (2%)	-
	Overhydration	2 (2%)	1 (1%)
Pulmonary	Apnea/hypoxia	3 (3%)	2 (2%)
Neurological	Cerebral infarction	1 (1%)	-
	Obturator neuropathy	1 (1%)	-
Other	Metabolic acidosis	2 (2%)	-
	Urinary leakage	1 (1%)	-
	Ureteroileal anastomosis stricture	1 (1%)	1 (1%)
	Postrenal failure	1 (1%)	1 (1%)
	Hyperkalemia	1 (1%)	-
	Hoarseness	1 (1%)	-
	Pseudogout	1 (1%)	-
	Liver dysfunction	1 (1%)	-

Among those with Grade ≥3 complications, three cases of ileus required nasogastric tube placement, and one case of UTI necessitated admission to the ICU. Additionally, two patients with wound infections required surgical drainage, and three patients with respiratory failure or fluid overload were managed with mechanical ventilation. Two cases of anastomotic stricture required nephrostomy for resolution.

Less frequent complications included deep vein thrombosis (n = 2), overhydration (n = 2, including one case requiring intervention), and a variety of metabolic, infectious, and gastrointestinal complications, each affecting a small number of patients.

Comparisons of complication rates between the low- and high-PNI groups for overall complications, severe complications, postoperative ileus, and postoperative infections are presented in Table 4. The low-PNI group had a significantly higher incidence of overall complications (19/27 (70%) vs. 31/76 (47%), p < 0.05) and postoperative ileus (13/27 (48%) vs. 14/76 (18%), p < 0.05) compared to the high-PNI group. Although the differences in severe complications and postoperative infections did not reach statistical significance, the low-PNI group exhibited higher rates of both outcomes than the high-PNI group.

**Table 4:** Overview of postoperative complications A p-value < 0.05 was considered statistically significant. CD, Clavien-Dindo; PNI, prognostic nutritional index

Variable	Total (n = 103), n (%)	PNI ≥45 (n = 76), n (%)	PNI <45 (n = 27), n (%)	p-value
All complications (CD I-V)	50 (48.5)	31 (41)	19 (70)	0.013
High-grade complications (CD III-V)	8 (8)	4 (5)	4 (15)	0.202
Postoperative ileus	28 (26.2)	14 (18)	13 (48)	0.005
Postoperative infection	22 (21.3)	15 (20)	6 (22)	0.586

Risk factors

Variables with p < 0.10 in univariate analysis - including age, sex, BMI, ASA-PS, CCI, clinical T and N stages, prior abdominal surgery, NAC, and type of urinary diversion - were entered into the multivariate logistic regression model. The multivariate analysis identified low PNI (p = 0.008, OR = 3.82) and intestinal urinary diversion (p = 0.033, OR = 3.33) as independent predictors of overall complications (Table 5). Similarly, low PNI (p = 0.008, OR = 3.90) and intestinal urinary diversion (p = 0.033, OR = 5.34) were independent predictors of postoperative ileus. No independent risk factors were identified for high-grade complications or postoperative infections.

**Table 5:** Multivariate analysis of complications after RARC Variables with p < 0.10 in univariate analysis were included in the multivariate logistic regression model. The univariate analysis evaluated PNI, age, sex, BMI, CCI, ASA-PS, clinical T and N stages, prior abdominal surgery, NAC, intestinal urinary diversion, and simultaneous nephrectomy. Statistical significance was defined as p < 0.05. ASA-PS, American Society of Anesthesiologists Physical Status; CD, Clavien-Dindo; CCI, Charlson Comorbidity Index; NAC, neoadjuvant chemotherapy; PNI, prognostic nutritional index; RARC, robot-assisted radical cystectomy

Postoperative outcomes	OR	95% CI	p-value
All complications (CD I-V)			
PNI <45	3.82	1.43-10.20	0.008
Intestinal diversion	3.33	1.11-10.00	0.033
High-grade complications (CD III-V)			
None	-	-	-
Postoperative ileus			
PNI <45	3.9	1.39 - 11.00	0.01
Intestinal diversion	5.34	1.06 - 26.70	0.042
Postoperative infection			
None	-	-	-

**Figure 1:**
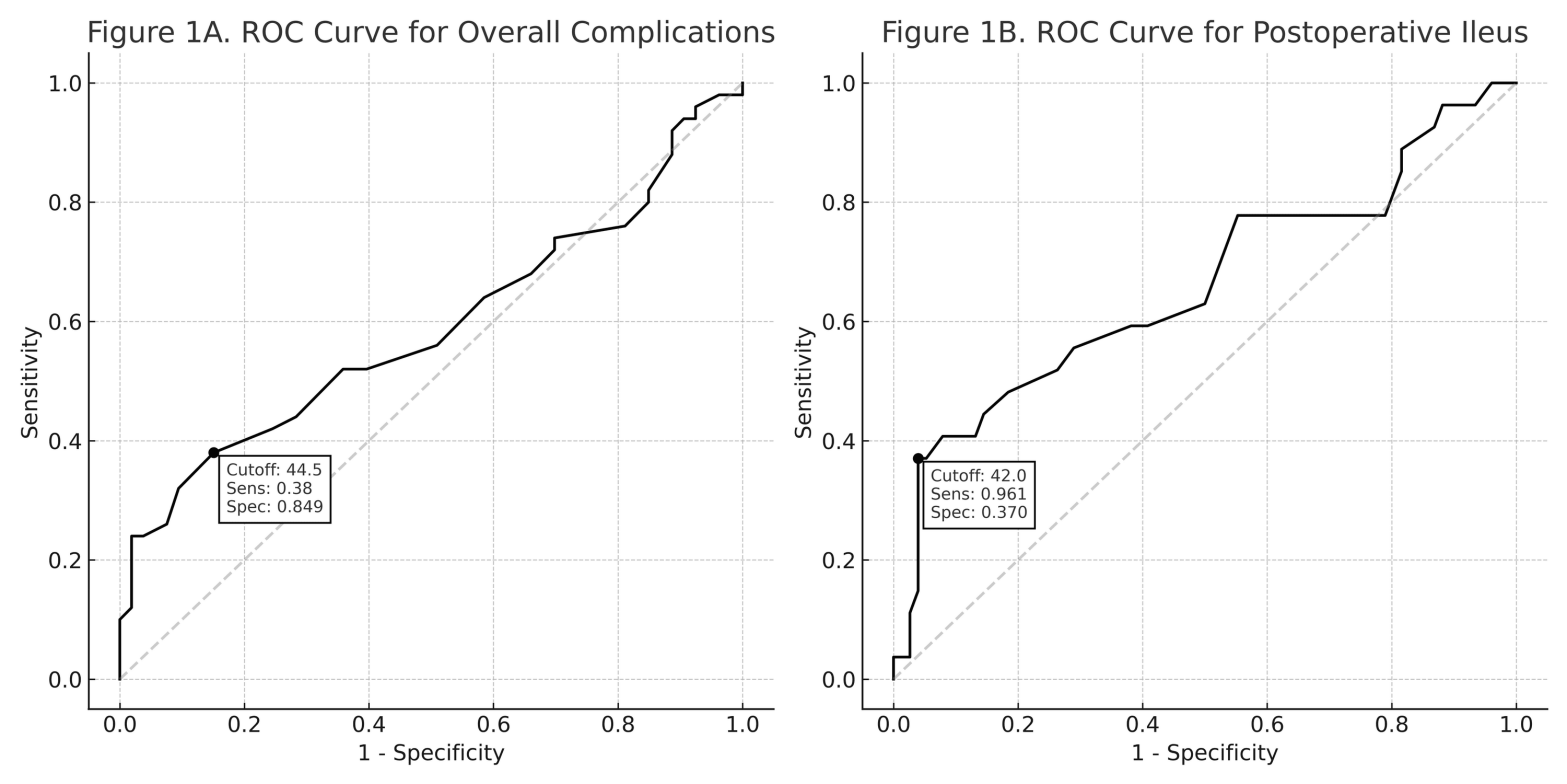
ROC curves of the PNI for predicting postoperative complications following RARC (A) ROC curve for overall complications, with an optimal cutoff value of 44.5 (sensitivity: 0.38, specificity: 0.85). (B) ROC curve for postoperative ileus, with an optimal cutoff value of 42.0 (sensitivity: 0.96, specificity: 0.37). The cutoff values shown represent statistically optimal thresholds determined by ROC analysis (Youden’s index) and may differ slightly from the clinically referenced cutoff value of 45 used elsewhere in the study. PNI, prognostic nutritional index; RARC, robot-assisted radical cystectomy; ROC, receiver operating characteristic

Receiver operating characteristic (ROC) curve analysis was performed to evaluate the predictive value of the PNI for postoperative complications. The area under the curve (AUC) was 0.68 for overall complications, with an optimal cutoff value of 44.5 (sensitivity: 0.38; specificity: 0.85). For postoperative ileus, the AUC was 0.74, with a cutoff of 42.0 (sensitivity: 0.96; specificity: 0.37). These findings indicate that the PNI has moderate discriminatory ability, particularly for predicting ileus (Figure 1). While a cutoff of 45 was used in the main analysis based on previous studies, the ROC analysis suggested optimal cutoff values of 44.5 for overall complications and 42.0 for postoperative ileus.

## Discussion

In this retrospective study, we examined the relationship between preoperative immunonutritional status, as assessed by the PNI, and postoperative complications in 103 patients who underwent RARC for bladder cancer. Our results revealed a significant association between a low PNI and an increased risk of overall complications within 30 days post-surgery. Notably, postoperative ileus was particularly prevalent among patients with low PNI, underscoring the importance of evaluating nutritional and immune status as part of perioperative risk assessment.

The link between low PNI and postoperative complications can be explained by its two components: serum albumin and lymphocyte count. Hypoalbuminemia reflects poor nutritional status and impairs collagen synthesis and tissue repair, while lymphopenia indicates compromised immune function. Together, these deficiencies increase vulnerability to surgical stress and postoperative complications.

Originally introduced by Onodera et al., the PNI is a well-established tool in surgical oncology [4]. Prior studies have demonstrated that a low PNI correlates with poorer outcomes in gastrointestinal and colorectal cancers [5,6]. In bladder cancer, Yu et al. found an association between low PNI and respiratory complications after cystectomy [7]. Building on these findings, our study identified a significant relationship between low PNI and postoperative ileus in patients undergoing RARC.

To evaluate the predictive accuracy of PNI, we performed ROC curve analysis. The AUC was 0.68 for overall complications and 0.74 for postoperative ileus, indicating moderate discriminatory ability. Although the optimal cutoff values derived statistically (44.5 and 42.0, respectively) differed slightly from the widely used threshold of 45, we chose to retain 45 for consistency with prior literature and clinical interpretability. This decision emphasizes generalizability over dataset-specific optimization, a trade-off we acknowledge as a limitation. Furthermore, while PNI may serve as a useful screening tool, its moderate AUC values suggest it should not be considered a definitive standalone predictor.

Postoperative ileus was the most common complication in our cohort, occurring in 26% of patients, consistent with previous reports ranging from 14.1% to 25.6% [17-19]. Defined by criteria from Khan et al. [12], ileus is a multifactorial condition influenced by bowel manipulation, nutritional deficiencies, and immune dysfunction. Our findings identify low PNI as a significant risk factor for postoperative ileus, with an AUC of 0.74 indicating moderate predictive ability. Although the lack of a standardized definition complicates comparisons across studies [20], other predictive tools have been investigated. For example, the CONUT score, which incorporates albumin, cholesterol, and lymphocyte count, was linked to ileus with an AUC of 0.58 and an OR of 2.91 [21]. The frailty-based Geriatric-8 score demonstrated an AUC of 0.716 and was also an independent predictor [19]. Compared to these, PNI offers a favorable balance of simplicity and predictive performance using routinely available laboratory data, making it a practical clinical option.

Intestinal urinary diversion has also been shown to independently contribute to postoperative complications, including ileus. Our findings support previous studies indicating that bowel manipulation during urinary diversion increases the risk of gastrointestinal dysfunction [22-24]. Although all diversions in our cohort were performed intracorporeally to minimize contamination and handling, this surgical step still carries inherent risks, especially in patients with low PNI. Enhanced perioperative management, including nutritional support, may help mitigate these risks.

Identifying high-risk patients via PNI assessments offers an opportunity for targeted interventions. Enhanced Recovery After Surgery (ERAS) protocols have demonstrated effectiveness in reducing complications and hospital stay length in bladder cancer surgery [25]. While our institution has partially implemented ERAS elements, a comprehensive protocol has yet to be adopted. Given the high incidence of ileus observed, incorporating preoperative oral nutritional supplementation and other measures may improve outcomes in at-risk patients. For example, Khaleel et al. reported that preoperative immunonutrition reduced infection-related complications in RARC patients [26]. Our findings suggest that integrating such interventions into routine care could enhance perioperative outcomes.

Limitations

This study has several limitations. As a retrospective analysis, it is subject to biases in data collection and complication reporting. The involvement of multiple surgeons may introduce variability, and the sample size is limited for subgroup and sensitivity analyses. Although we applied a standardized ileus definition, inconsistencies across studies remain. Inflammatory markers such as CRP or IL-6 were not routinely measured; since these can influence serum albumin and lymphocyte counts, their absence may reduce the interpretability of PNI and confound its association with complications. Future studies should include concurrent inflammatory status assessments to enhance immunonutritional evaluations’ accuracy. Additionally, individualized nutritional interventions for low-PNI patients were not routinely performed during the study period, limiting clinical applicability. It remains unclear whether identifying high-risk patients through PNI screening alone would improve outcomes without targeted interventions. Based on these results, our institution has since adopted active nutritional support policies for preoperatively malnourished patients undergoing RARC, the effectiveness of which warrants prospective evaluation.

Finally, we did not conduct subgroup analyses based on key characteristics such as urinary diversion type, age, or comorbidity burden due to the limited sample size, which could lead to underpowered or misleading conclusions. However, the predictive value of PNI may vary in specific high-risk populations (e.g., elderly patients or those with high CCI scores), representing an important direction for future research. These considerations highlight the need for larger, multi-institutional prospective studies to confirm and extend our findings.

## Conclusions

This study demonstrated that assessing nutritional and immunological status before surgery offers valuable insight into the risk of complications in patients undergoing RARC for bladder cancer. This status can be affected by several factors commonly seen in cancer patients, such as chronic inflammation, increased metabolic demands, chemotherapy-induced immunosuppression, and reduced oral intake. A simple evaluation of preoperative nutritional markers enables clinicians to identify high-risk individuals who may benefit from personalized perioperative management strategies. Future research should focus on the effectiveness of interventions like ERAS protocols and targeted nutritional or immunological support to enhance surgical outcomes and reduce complication rates in this population.
